# Contribution of inter-trial phase coherence at theta, alpha, and beta frequencies in auditory change detection

**DOI:** 10.3389/fnins.2023.1224479

**Published:** 2023-11-09

**Authors:** Caifeng Xia, Jinhong Li, Rong Yan, Wenwen Su, Yuhe Liu

**Affiliations:** ^1^Department of Otolaryngology Head and Neck Surgery, Peking University First Hospital, Beijing, China; ^2^State Key Laboratory of Cognitive Neuroscience and Learning, Beijing Normal University, Beijing, China; ^3^School of Systems Science, Beijing Normal University, Beijing, China; ^4^Department of Otolaryngology Head and Neck Surgery, Beijing Friendship Hospital, Capital Medical University, Beijing, China

**Keywords:** auditory change detection, inter-trial phase coherence (ITC), theta rhythm, alpha rhythm, beta rhythm

## Abstract

**Introduction:**

Auditory change detection is a pre-attentive cortical auditory processing ability. Many neurological and psychological disorders can lead to defects in this process. Some studies have shown that phase synchronization may be related to auditory discrimination. However, the specific contributions of phase synchronization at different frequencies remain unclear.

**Methods:**

We analyzed the electroencephalogram (EEG) data of 29 healthy adults using an oddball paradigm consisting of a standard stimulus and five deviant stimuli with varying frequency modulation patterns, including midpoint frequency transitions and linear frequency modulation. We then compared the peak amplitude and latency of inter-trial phase coherence (ITC) at the theta(θ), alpha(α), and beta(β) frequencies, as well as the N1 component, and their relationships with stimulus changes. At the same time, the characteristics of inter-trial phase coherence in response to the pure tone stimulation and chirp sound with a fine time-frequency structure were also assessed.

**Result:**

When the stimulus frequency did not change relative to the standard stimulus, the peak latency of phase coherence at β and α frequencies was consistent with that of the N1 component. The inter-trial phase coherence at β frequency (β-ITC)served as a faster indicator for detecting frequency transition when the stimulus frequency was changed relative to the standard stimulus. β-ITC demonstrates temporal stability when detecting pure sinusoidal tones and their frequency changes, and is less susceptible to interference from other neural activities. The phase coherence at θ frequency could integrate the frequency and temporal characteristics of deviant into a single representation, which can be compared with the memory trace formed by the standard stimulus, thus effectively identifying auditory changes. Pure sinusoidal tone stimulation could induce higher inter-trial phase coherence in a smaller time window, but chirp sounds with a fine time-frequency structure required longer latencies to achieve phase coherence.

**Conclusion:**

Phase coherence at theta, alpha, and beta frequencies are all involved in auditory change detection, but play different roles in this automatic process. Complex time-frequency modulated stimuli require longer processing time for effective change detection.

## 1. Introduction

The auditory system constantly monitors the surrounding environment, enabling the timely detection of changes in ambient sounds and allocating cognitive resources to identify potential dangers, which is crucial for survival. For example, this ability allows individuals to detect the sound of a predator approaching from behind. Consequently, the auditory system is often referred to as an early warning system (Dalton and Lavie, 2004). The automatic auditory change detection process does not require attention (Sams et al., 1985) and is an embodiment of primitive intelligence (Naatanen et al., 2001).

Mismatch negativity (MMN), a component of auditory event-related potential (AERP) is a neural marker commonly used for evaluating auditory discrimination. MMN is commonly obtained under an oddball paradigm, which consists of repeated standard stimuli and infrequently deviant stimuli (Mäntysalo and Näätänen, 1987). Deviant stimuli differ from the standard stimuli in terms of physical features (e.g., frequency, duration, location, etc.) or high-level abstract rules that violate the memory trace formed by the standard stimuli, thus inducing MMN, which peaks 100–250 ms after the deviant stimuli are presented (Rosburg et al., 2005; Näätänen et al., 2011). An increasing degree of sound deviance results in a larger MMN amplitude and shorter latency, often resulting in significant overlap with the N1 component (Tiitinen et al., 1994; May and Tiitinen, 2010).

Mismatch negativity is an extensively researched neural indicator known to manifest in response to various types of auditory change, including sound omissions (Näätänen et al., 2005), complex stimuli deviations, and even complex rule deviations (Tervaniemi et al., 1994). Nevertheless, there exists an alternative perspective based on the adaptation hypothesis, suggesting that MMN fundamentally signifies a subtractive process (May and Tiitinen, 2010). This proposition implies that the initial encoding of stimulus distinctions through the N1 could potentially account for the observed variations in human ERPs without necessitating the engagement of higher order cognitive processes (Fitzgerald and Todd, 2020). When interpreting differences in the physical characteristics of sound eliciting distinct N1 responses from the perspective of adaptation hypothesis, certain challenges arise. N1 exhibits a broad latency range when representing stimuli with a fine time-frequency structure, making it difficult to distinguish from MMN. Additionally, in ongoing sound sequences, the neural activity induced by previously presented sound stimuli can influence the N1 elicited by subsequent sound stimuli.

With the advancement of EEG data analysis technology, time-frequency analysis provides more information about activities at different neural frequencies. In contrast to event-related potentials, time-frequency analysis quantifies the variability of neural responses in phase across trials, and the consistency of neural response timing at specific frequencies and time points relative to experimental events (Buzsaki and Draguhn, 2004; Canolty and Knight, 2010; Euler et al., 2015). Phase changes can be described by the inter-trial phase coherence (ITC or ITPC), also known as the phase-locking value (PLV), which ranges from zero to one. ITC values closer to one indicate stronger phase consistency within a specific frequency band at that time point (Varela et al., 2001; van Diepen and Mazaheri, 2018). Further, ITC provides information on the overall consistency of neural responses during an experiment, potentially offering a more reliable method for assessing neural reliability (Buzsaki and Draguhn, 2004; Canolty and Knight, 2010; Ding and Simon, 2013).

Several studies have explored the role of ITC in different frequency bands during the auditory discrimination process. In a magnetoencephalography (MEG) study, Hsiao et al. (2009) found that phase-locked θ and α oscillations are related to auditory change detection represented by MMN. Fuentemilla et al. (2008) posited that the frontal component of MMN is generated through power modulation at θ frequency, while the temporal component is primarily produced by phase resetting. By comparing the oddball paradigm with the control sequence, Ko et al. (2012) found that the additional phase resetting and power modulation at the θ and α frequencies induced by the deviant stimulus were related to auditory change detection, rather than to the differences in physical characteristics between the standard and deviant stimuli. In addition, Bishop and Hardiman (2010) investigated MMN measurement methods within individuals and found that ITC was a more reliable indicator of MMN than ERSP. Even in some individuals who could behaviorally differentiate deviant stimuli but did not elicit a clear MMN, changes in the ITC were observed (Bishop and Hardiman, 2010). All these studies employed the most common method for comparing the response differences between deviant and standard stimuli, which involves subtracting the average phase coherence strength of the standard from that of the deviant (Alho, 1995). However, this approach may lead to the loss of information at higher frequencies where strong phase synchronization is more difficult to achieve, which could explain why most current findings primarily focus on the θ and low α frequencies. Furthermore, the pattern of changes in the deviants in these studies was relatively simple, and the phase coherence at different frequencies, such as θ and α, appeared to exhibit similar detection roles for sound deviations. However, efficient automated information-processing systems do not appear to require many equal functional roles to accomplish the same task. Therefore, we hypothesize that in more complex acoustic scene analyses, the phase coherence at different frequencies may play different roles in detecting auditory change.

In this study, we designed an oddball sequence consisting of one standard and five deviant stimuli, each exhibiting different internal change patterns in the frequency dimension, such as midpoint transition and linear modulation. These patterns mirror real-world sound variations like the dynamic pitch changes in musical melodies from keyboard instruments and the continuous glissando in string instruments, which can be perceptually experienced. We analyzed the variation in phase coherence with time at different frequencies and attempted to illustrate the role of different frequency bands in the detection of auditory changes. Besides, we compared these findings with the properties of N1/N1-like responses.

## 2. Materials and methods

### 2.1. Participants

A total of 34 right-handed healthy individuals (16 females, aged 23–34 years, mean 28.4 years) participated in this study, and their hearing sensitivity was confirmed to be normal by pure tone audiometry. This study was approved by the Ethics Committee of Peking University First Hospital, and each participant provided written informed consent.

### 2.2. Stimuli and procedure

**FIGURE 1:**
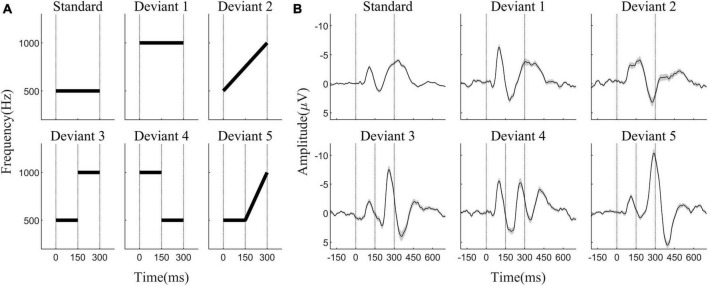
**(A)** The spectrogram of six stimuli. The duration of all stimuli was 300 ms; SS and DS1 are pure sinusoidal tones, with SS at 500 Hz and DS1 at 1000 Hz, and the frequencies of DS2 to DS5 change dynamically within the stimuli. The onset, mid-point transition point, and offset of the stimulus are indicated by vertical dashed lines. **(B)** Averaged auditory event-related potential waveforms from all 29 participants in response to the six stimuli at electrode Fz. After a mid-point transition, the sound attribute changed (DS3, DS4, DS5), resulting in the elicitation of a negative wave resembling N1, which occurred approximately in the 250 to 300 ms range and was termed as N1-like.

The oddball paradigm consisted of one standard and five deviant stimuli. Figure 1A shows the spectrogram of each stimulus. All auditory stimuli had a duration of 300 ms with rise and fall times of 5 ms. The standard stimulus (SS) was a 500 Hz pure sinusoidal tone. The five deviant stimuli were as follows: deviant stimulus 1 (DS1), a 1000 Hz pure sinusoidal tone; deviant stimulus 2 (DS2): linear sweep from 150 to 300 Hz within 300 ms. This is a chirp signal with a fine time-frequency structure compared to a simple sinusoidal tone; deviant stimulus 3 (DS3): a 150 ms 500 Hz pure tone (DS3-1) followed by a 150 ms 1000 Hz tone (DS3-2); this stimulus exhibited a prominent frequency transition at its midpoint, with the first segment identical to the standard stimulus; deviant stimulus 4 (DS4): a 150 ms 1000 Hz pure tone (DS4-1) followed by a 150 ms 500 Hz tone (DS4-2), with an opposite frequency transition direction compared to DS3; and deviant stimulus 5 (DS5): a 150 ms 500 Hz pure tone (DS5-1) followed by a 150 ms chirp signal sweeping linearly from 500 to 1000 Hz (DS5-2). All the stimuli were generated using MATLAB (R2020b).

A total of 800 trials were presented, with a ratio of 480:64:64:64:64:64 between the standard and five deviants. The overall ratio of standard to deviant stimuli was 6:4. The ISI between adjacent stimuli varied randomly between 700 and 1000 ms. The first 15 trials consisted of only the SS, after which the stimuli were presented randomly. At least one SS was presented between two deviants. The duration of the test was approximately 15 min.

Participants sat in a soundproof room, and stimuli were played at 70 dB from a stereo directly in front of them, one meter away. The participants were instructed to close their eyes and relax while completing the passive auditory experiment, ignoring sound stimuli and providing no response. During this time, the EEG signals were recorded.

### 2.3. EEG recording

Nineteen Ag/AgCl electrodes were placed on the scalp according to the international 10-20 electrode positioning system, with electrode positions at Fp1, Fp2, Fz, F3, F4, FCz, FC3, FC4, Cz, C3, C4, FT7, FT8, P_z_, P3, P4, TP7, TP8, and Oz, as well as on the two mastoids (Left: Lm and Right: Rm). All the electrodes were referenced online to the right mastoid. Recordings were obtained using Blackrock Microsystems Technology (Salt Lake City, UT, USA). Channels were amplified, filtered between 0.3 and 2000 Hz, and digitized at 10 kHz.

### 2.4. Data analysis

The EEG signals were preprocessed using the EEGLAB toolbox and custom MATLAB scripts (Delorme and Makeig, 2004). The signal was down-sampled to a rate of 1000 Hz, followed by the application of a 1–45 Hz bandpass filter. Data were subsequently re-referenced to the average of the two mastoids. After visually inspecting the signals to identify and remove corrupted signal segments, independent component analysis (ICA) was performed to eliminate eye movement and muscle artifacts. Five participants were excluded from further analysis due to excessive artifacts (two participants) and the inability to observe the N1 component (three participants). Data from the remaining 29 participants (16 females, aged 23–32 years, mean 27.9 years) were used for subsequent analyses.

The signal was segmented relative to the stimulus onset (−300 to 800 ms), with a 300 ms pre-stimulus interval used as a baseline. The epochs with signal ranges exceeding 100 mv in any channel were excluded. We obtained the average time-domain signal for each participant across all trials for each stimulus. The N1 peak amplitudes and latencies of the Fz electrode were analyzed. The negative peak within the 50–160 ms time window was identified as N1 in SS, whereas for DS2, the time window was expanded to 50–300 ms. The N1 of the second segments of DS3–DS5 was referred to as N1-like. To define N1-like, DS 3–2 and 4–2 had a time window of 200–310 ms, while for DS5-2, the time window was 200–450 ms.

We calculated the ITC using complex Morlet wavelet-based spectral decomposition (VanRullen, 2016; Do Carmo-Blanco et al., 2022). Three hundred linearly spaced frequencies ranging from 1 to 40 Hz were analyzed. The number of cycles in the wavelet increased from three to six according to the frequency. To maintain consistency with the deviant stimulus, we randomly chose 64 epochs for the standard stimulus because the ITC is sensitive to the number of trials. We averaged the ITC value in the frequency dimension of 4–8 Hz at every time point to represent the θ-band ITC (θ-ITC). The ITC value averaged between 8 and 12 Hz represented the α-band ITC (α-ITC), and between 12 and 18 Hz represented the beta-band ITC (β-ITC). We obtained the Event-Related Spectral Perturbation (ERSP) using the same wavelet decomposition parameters. We applied a logarithmic transformation and selected a baseline time window from −250 to −50 ms before the onset of each trial as a correction step. The decibel-normalized power was then employed for further analysis. Similarly, the corresponding ERSP values were averaged at each timespoints for the θ-band (θ-ERSP), α-band (α-ERSP), and β-band (β-ERSP) within their respective frequency ranges. We analyzed the peak amplitudes and latencies of ITC and ERSP for the three frequencies at the Fz electrode.

We analyzed the amplitude and latency differences among various neural signal indices, including N1/N1-like, θ-ITC, α-ITC, β-ITC, and θ-ERSP, in response to standard and deviant auditory stimuli. We conducted a one-way repeated ANOVA to examine the latency difference among different neural signal indices, where the main effect was the indices. Similarly, we compared responses of the same signal indices to different auditory stimuli using another one-way rANOVA, with a focus on the main effect of the stimulus type. Upon rejection of the null hypothesis following rANOVA, we provided effect size ($\eta_{\text{p}}^{2}$) values. To further assess the differences between each level, both the Bonferroni’s *post-hoc* test and a permutation paired *t*-test were employed. For specific pairwise comparisons, we employed permutation-paired *t*-tests. In the permutation paired *t*-test, the null hypothesis stated that there was no significant difference between the two conditions. To test this hypothesis, we first calculated the *t*-values of the original paired data. Next, the labels of the paired data points were randomly shuffled or permuted, and the *t*-value was recalculated for the permuted data. This randomization and *t*-value calculation process was repeated 10,000 times to generate a distribution of permuted *t*-values. The original *t*-value was then compared with the distribution of the permuted *t*-values to determine its position within the distribution, expressed as a *z*-value. The corresponding probability (*p*-value) was calculated and was denoted as *p*_z_. Statistical significance (*p*_z_) was set as *p* < 0.05. The null hypothesis was rejected, indicating a significant difference between the two conditions. When comparing the latencies between the first and the second segments, we adjusted the latency of the second segment by subtracting 150 ms before making the comparison. The aforementioned statistical processes were performed using the Statistical Package for the Social Sciences (SPSS 25.0) and MATLAB (R2020b).

## 3. Results

**FIGURE 2:**
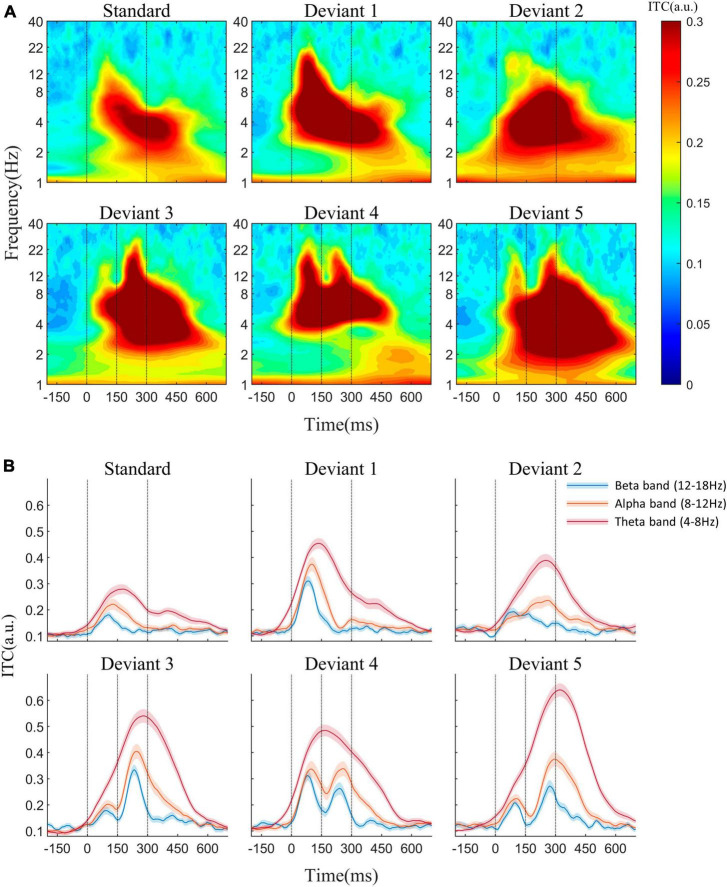
**(A)** Inter-trial phase coherence (ITC) in response to the standard and five deviant stimuli at the Fz electrode. **(B)** ITC waveforms of theta, alpha, and beta frequencies. When stimuli presented a single auditory attribute (SS, DS1, and DS2), α-ITC and β-ITC showed unimodal dynamic processes. However, in the presence of a frequency transition in the middle of the trial (DS3, DS4, and DS5), they exhibited bimodal dynamic processes. Across all the stimuli, the θ-ITC exhibited a unimodal dynamic process.

**FIGURE 3:**
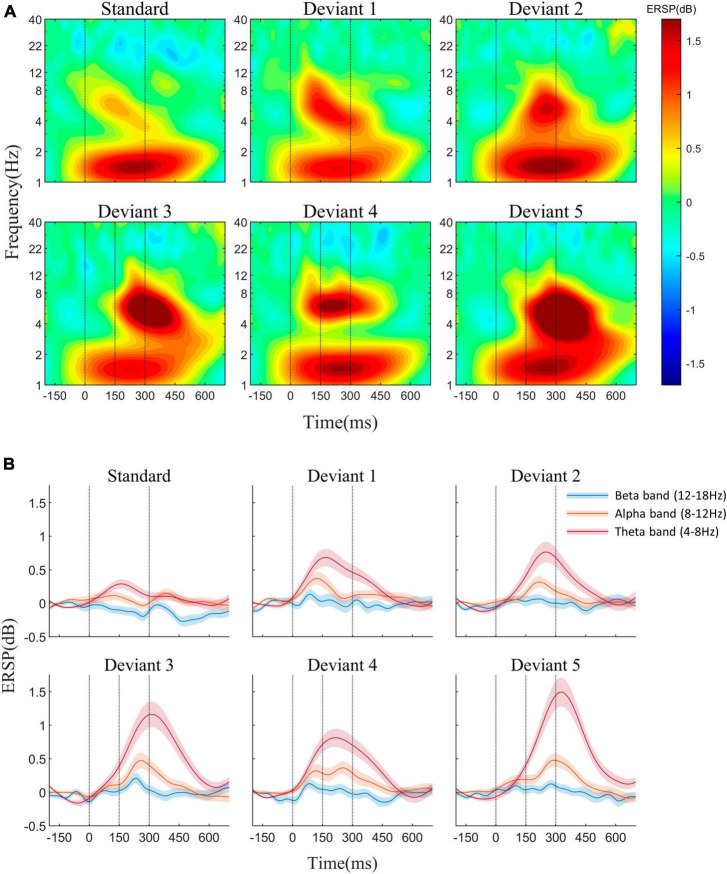
**(A)** Event-related spectral perturbation (ERSP) in response to the standard and five deviant stimuli at the Fz electrode. **(B)** ERSP waveforms of theta, alpha, and beta frequencies. β-ERSP exhibited no significant response, while α-ERSP showed a weak response, with its outcome being based on the group average.

The average AERP waveforms of the 29 participants are shown in Figure 1B. The ITC results in response to the six auditory stimuli are presented in Figure 2A. Waveform plots were used to demonstrate the dynamic temporal features of phase coherence across the three frequency bands (θ-ITC, α-ITC, and β-ITC), as illustrated in Figure 2B. Across all the stimuli, the θ-ITC exhibited a unimodal dynamic process. α-ITC, β-ITC and the time-domain N1 displayed unimodal dynamic processes when the stimuli presented a single auditory attribute (SS, DS1, and DS2). In contrast, they exhibited bimodal dynamic processes when a frequency transition occurred in the middle of the trial (DS3, DS4, and DS5). The latencies of N1/N1-like and θ-ITC, α-ITC, and β-ITC for the six stimuli are listed in Table 1, while their amplitude intensities are provided in Table 2. The ERSP results are presented in Figure 3.

**TABLE 1:** Mean latency (ms) of N1/N1-like and ITC at θ, α, and β frequencies in response to the six stimuli at the Fz electrode (Standard errors are reported in parentheses).

		N1/N1 like	β-ITC	α-ITC	θ-ITC
SS		105.72 (13.89)	101.62 (23.15)	109.59 (54.86)	183.59 (54.86)
DS1		100.41 (9.45)	84.38 (13.28)	102.41 (14.73)	140.00 (32.53)
DS2		149.14 (31.60)	114.24 (45.80)	157.90 (61.71)	246.79 (59.86)
DS3	DS3-1	106.83 (16.58)	95.10 (21.09)	105.48 (24.68)	288.72 (50.35)
	DS3-2	263.34 (12.13)	237.38 (18.75)	245.24 (14.57)	
DS4	DS4-1	100.24 (11.78)	84.14 (13.96)	99.83 (20.31)	188.21 (69.68)
	DS4-2	264.48 (14.46)	242.41 (20.27)	254.43 (22.34)	
DS5	DS5-1	107.79 (15.92)	99.79 (21.13)	100.25 (24.04)	314.28 (42.73)
	DS5-2	293.79 (14.92)	275.69 (34.95)	294.00 (30.04)	

SS, standard stimuli; DS, deviant stimuli; ITC, inter-trial phase coherence.

**TABLE 2:** Mean amplitude (μV/a.u.) of N1/N1-like and ITC at θ, α, and β frequencies in response to the six stimuli at the Fz electrode (Standard errors are reported in parentheses).

		N1/N1 like	β-ITC	α-ITC	θ-ITC
SS		−3.53 (1.67)	0.20 (0.06)	0.24 (0.11)	0.32 (0.09)
DS1		−6.99 (2.69)	0.33 (0.11)	0.38 (0.15)	0.47 (0.11)
DS2		−6.13 (2.89)	0.25 (0.09)	0.29 (0.12)	0.42 (0.13)
DS3	DS3-1	−3.01 (2.38)	0.21 (0.08)	0.22 (0.09)	0.57 (0.13)
	DS3-2	−8.29 (3.91)	0.36 (0.11)	0.42 (0.15)	
DS4	DS4-1	−6.34 (2.43)	0.34 (0.10)	0.35 (0.17)	0.52 (0.10)
	DS4-2	−5.99 (4.36)	0.29 (0.12)	0.36 (0.15)	
DS5	DS5-1	−3.77 (2.04)	0.23 (0.07)	0.25 (0.10)	0.65 (0.14)
	DS5-2	−11.29 (3.67)	0.32 (0.12)	0.40 (0.15)	

SS, standard stimuli; DS, deviant stimuli; ITC, inter-trial phase coherence.

### 3.1. Comparison of N1/N1-like with α-ITC and β-ITC

**FIGURE 4:**
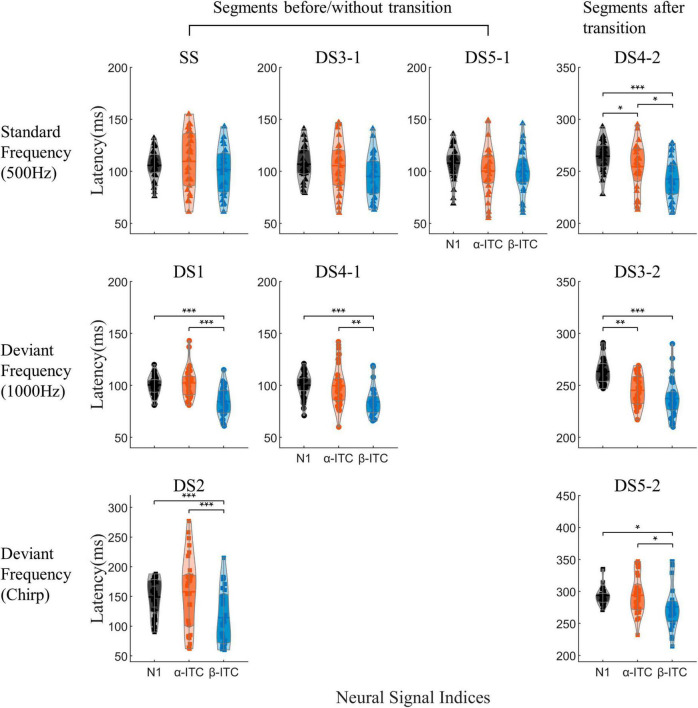
The peak latencies of N1, α-ITC, and β-ITC are depicted for each auditory stimulus. Mean values and quartiles are annotated within the violin plots. The *y*-axis coordinates for the “Segments after transition” section have been vertically shifted by an additional 150 ms. β-ITC functioned as a more rapid indicator for detecting sound frequency changes, whereas N1 and α-ITC exhibited consistent peak latencies. *p_z_ < 0.05, **p_z_ < 0.01,***p_z_ < 0.001.

β-ITC served as a faster indicator for detecting changes in sound frequency, whereas N1 and α-ITC exhibited consistent peak latencies (Figure 4). When the sound frequency did not change (SS, DS3-1, and DS5-1), there were no significant differences in the latencies of the three neural signal indices [main effect of three indices, SS: *F*_(2,56)_ = 1.314, *p* = 0.277; DS3-1: *F*_(2,56)_ = 3.045, *p* = 0.056; DS5-1: *F*_(2,56)_ = 2.209, *p* = 0.12]. While when the sound frequency was changed relative to the standard stimulus (Figure 4, bottom two rows, DS1, DS2, DS3-2, DS4-1, and DS5-2), the latencies of the three neural signal indices were significantly different [DS1: *F*_(2,56)_ = 25.191, *p* < 0.001, $\eta_{\text{p}}^{2}$ = 0.4736; DS2: *F*_(2,56)_ = 10.780, *p* = 0.001, $\eta_{\text{p}}^{2}$ = 0.278; DS3-2: *F*_(2,56)_ = 23.152, *p* < 0.001, $\eta_{\text{p}}^{2}$ = 0.4526; DS4-1: *F*_(2,56)_ = 12.9, *p* < 0.001, $\eta_{\text{p}}^{2}$ = 0.3233; DS5-2: *F*_(2,56)_ = 4.158, *p* = 0.0207, $\eta_{\text{p}}^{2}$ = 0.1293]. The latency of β-ITC was shorter than that of the N1/N1-like (all *p*_z_ < 0.05) in those stimuli. In DS3-2 and DS4-2, α-ITC reached its peak faster than N1-like (all *p*_z_ < 0.05), whereas, in other stimuli, there were no significant differences in latency between α-ITC and N1, indicating that N1-like was contaminated by the preceding stimulus.

**FIGURE 5:**
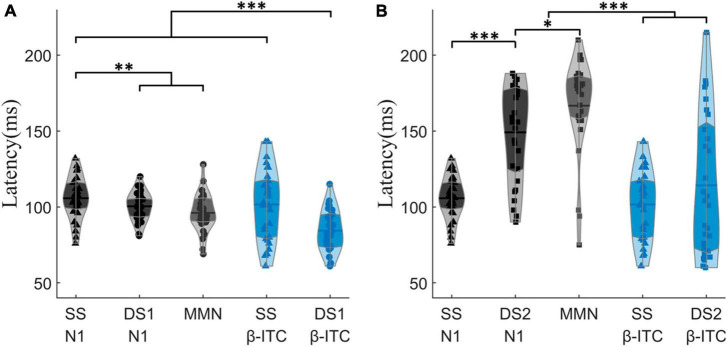
Comparison of N1, MMN, and β-ITC peak latencies between SS, DS1, and DS2. In **(A)**, DS1 exhibits a significantly shorter latency of β-ITC when detecting pure sinusoidal deviants. In **(B)**, when detecting chirp stimuli with fine time-frequency structures, DS2 does not show a shorter latency in β-ITC compared to SS. *p_z_ < 0.05, **p_z_ < 0.01, ***p_z_ < 0.001.

Specifically, β-ITC has a significantly faster latency than N1 and MMN when presented with pure sinusoidal tone deviations (Figure 5A). The latency of N1 in SS is 105.72 ± 13.89 ms, while in DS1, it is 100.41 ± 9.45 ms, which is significantly faster than the latency in SS (*p*_z_ = 0.009). The latency of MMN in DS1 is 96.07 ± 12.72 ms. In SS, the latency of β-ITC is 101.62 ± 23.15 ms, which does not differ from the latency of N1 (*p*_z_ = 0.431). However, in DS1, the latency of β-ITC is 84.38 ± 13.28 ms, which is significantly faster than the latency of β-ITC in SS (*p*_z_ < 0.001), significantly faster than the latency of N1 in DS1 (*p*_z_ < 0.001), and also significantly faster than the classic difference detection indices MMN (*p*_z_ < 0.001).

When a fine structural chirp sound was presented, the latency of N1 was prolonged compared to the standard stimulus (SS vs. DS2: *p*_z_ < 0.001). However, the latency of β-ITC did not show significant changes, nor did it exhibit the phenomenon of a quickened latency similar to DS1 (Figure 5B). Notably, the amplitude of β-ITC in the chirp sound was significantly higher than that in the standard stimulus (SS vs. DS2: *p*_z_ = 0.009), but much lower than that in the deviant pure tone stimulus (DS1 vs. DS2: *p*_z_ < 0.001) (as seen in Figure 1).

**FIGURE 6:**
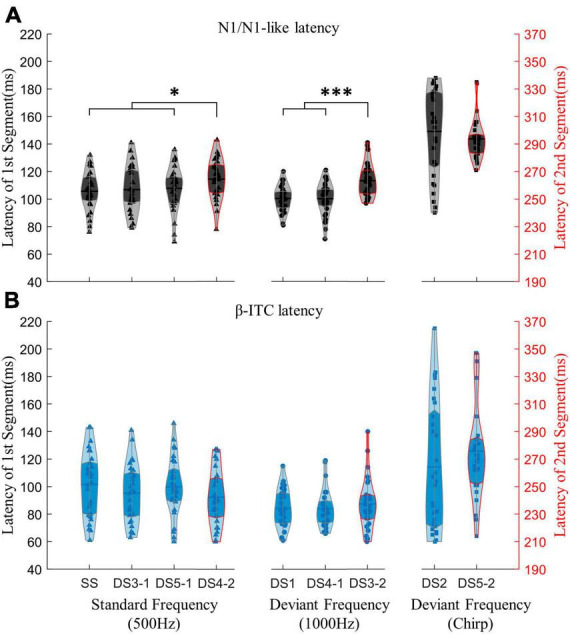
Comparing the impact of the mid-point transition on the latency of N1/N1-like and β-ITC. Results under the “segment after transition” condition are outlined in red on the violin plot, with the corresponding time axis on the right shifted by an additional 150 ms. In **(A)**, the latency of N1-like following the transition point is significantly extended due to the neural activity induced by stimuli preceding the transition. In **(B)**, β-ITC remains unaffected in latency when responding to pure sinusoidal tones and detecting their changes. Stimuli with fine time-frequency structures (chirp sounds in this experiment) result in prolonged latencies for both N1/N1-like and β-ITC. *p_z_ < 0.05, ***p_z_ < 0.001.

Importantly, when processing pure sinusoidal tones or detecting pure sinusoidal tone frequency changes, β-ITC is a stable indicator in the time dimension, and its latency is not affected by the stimulus before the mid-point transition (Figure 6B). The latency of N1-like is affected by the neural activity of stimulus before the transition, resulting in slower latency (Figure 6A). When the presented stimulus is a standard frequency, the latency of N1-like component after the midpoint transition slows down (DS4-2 vs. SS, DS3-1, DS5-1, all *p*_z_ < 0.05). When the presented stimulus is a deviant sinusoidal tone, the latency of N1-like does not show faster latency as N1 does, but also shows slower latency (DS3-2 vs. DS1, DS4-1, all *p*_z_ < 0.001). In chirp sounds, due to the fine time-frequency structure, the latency of both N1 and β-ITC slows down, so that the influence of neural response before the transition point is indistinguishable. Neither α-ITC, β-ITC, nor N1 changed significantly between stimuli with the same stimulation frequency but different duration (300 or 150 ms). There were no significant differences in N1 amplitude and latency for stimuli beginning with a 1000 Hz stimulus (DS1 vs. DS4-1, amplitude: *p*_z_ = 0.2247, latency: *p*_z_ = 0.8891). Similarly, there were no significant differences in N1 latency and amplitude for stimuli beginning with a 500 Hz stimulus [SS and DS3-1, DS5-1, amplitude: *F*_(2,56)_ = 3.437, *p* = 0.039, $\eta_{\text{p}}^{2}$ = 0.109, *post-hoc* test not significant, latency: *F*_(2,56)_ = 0.381, *p* = 0.685]. Consistent results were found in α-ITC and β-ITC (all *p*_z_ > 0.05).

### 3.2. Results of ERSP and θ-ITC

**FIGURE 7:**
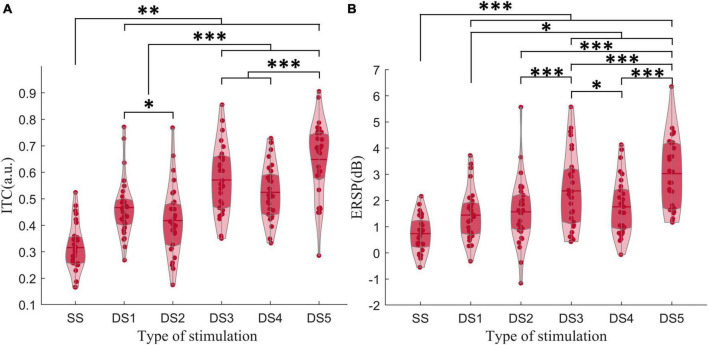
**(A)** The amplitude of θ-ITC. **(B)** The amplitude of θ-ERSP. When a frequency change occurred after the mid-point transition, the β-ITC amplitude was significantly greater than when the frequency change occurred at the onset. *p_z_ < 0.05, **p_z_ < 0.01, ***p_z_ < 0.001.

In the ERSP results, β-ERSP did not show obvious response, α-ERSP showed weak response, but its result was based on group average. The peak of α-ERSP had a large range of variation among individuals, and some individuals did not show obvious peak response. θ-ERSP and θ-ITC both showed unimodal dynamic process, even if frequency transition occurred in the stimulus (Figure 3). The peak amplitude of θ-ITC for all deviant stimuli was significantly higher than that for the standard stimulus (all *p*_z_ < 0.01). We found that when frequency change occurred after the mid-point transition, the β-ITC amplitude was significantly greater than when frequency change occurred at the onset (DS3 vs. DS1: *p*_z_ < 0.001, DS5 vs. DS2: *p*_z_ < 0.001). θ-ERSP showed consistent results (Figure 7). Lastly, a comparison between the pure sinusoidal tone and chirp stimulus variations revealed a noticeable prolongation of the θ-ITC latency (DS1 vs. DS2: *p*_z_ < 0.001, DS3 vs. DS5: *p*_z_ < 0.001).

## 4. Discussion

Our study focused on the dynamic changes of ITC at θ, α, and β frequencies in the oddball sequence over time. We found that the phase coherence at all three frequencies could detect auditory changes. θ-ITC exhibited a unimodal response in all stimuli, whereas the stimuli with midpoint frequency change (DS3, DS4, and DS5) could induce a bimodal response at α and β frequencies. Additionally, β-ITC demonstrated a faster response to auditory change compared to α-ITC and N1. β-ITC demonstrated temporal stability when detecting pure sinusoidal tones and their frequency changes, and is less susceptible to interference from other neural activities. Chirp sounds with a fine time-frequency structure required longer latencies to achieve phase coherence.

### 4.1. Characteristics of ITC at α and β frequencies in auditory change detection

Neural oscillations at the α and β frequencies are generally believed to represent the activation of inhibitory neurons in brain areas processing task-irrelevant information, so-called control by inhibition (Klimesch et al., 2007; Jensen and Mazaheri, 2010; Waldhauser et al., 2012). However, recent studies have discovered that α and β oscillations also participate in the bottom-up feedback regulation of sensory predictions (Hillebrand et al., 2016; Suda et al., 2022), which is one of the theoretical models explaining auditory change detection. Our study found that when the stimulus frequency did not change (SS, DS3-1, and DS5-1), the latency of α-ITC and β-ITC peak coincided with the N1 component of the AERP. It is well known that the N1 generator encodes stimulus information only for the first 40–50 ms after stimulus onset, which prevents N1 from discriminating differences in stimulus duration beyond this time range (Banai et al., 2007; Johnson et al., 2007; Näätänen et al., 2011). In our study, the ITC at α and β frequency also failed to discriminate these stimuli with the same frequency but different durations (SS, DS3-1, and DS5-1). The consistency of latency and response characteristics suggest that α and β phase modulation may be involved in the generation of the N1 component. Previous studies have also found that the α-ITC is related to changes in early ERP components in some auditory cognitive processes. Koerner and Zhang (2015) observed that trial-by-trial changes in α band phase coherence could predict changes in the N1–P2 complex induced by noise in a speech recognition task. Similarly, Klimesch et al. also observed that α band phase coherence was related to the generation of the P1–N1 complex in a visual memory task (Klimesch et al., 2004).

The essence of the oddball paradigm is that the frequencies of standard and deviant stimuli are different, leading to different refractoriness (Yabe et al., 1998; May and Tiitinen, 2010). In the time-domain analysis, the adaptation hypothesis suggests that MMN is the N1 difference between the deviant and the standard stimuli (Ulanovsky et al., 2003; Jaaskelainen et al., 2004). In our study, α-ITC and β-ITC showed adaption similar to those of N1. The peaks of α-ITC and β-ITC for the repeated standard frequency (500 Hz) were significantly lower than those for infrequent deviant stimuli (DS1, DS2, DS3-2, DS4-1, and DS5-2). Comparing DS3-1 and DS4-2, both of them have a standard pitch (500 Hz) and a duration of 150 ms. However, due to its occurrence after the mid-point transition, DS4-2 exhibits stronger amplitudes in both β-ITC and α-ITC. This suggests that the changes in the peak amplitude of ITC at α and β frequencies were not caused by stimulus frequency differences but were likely due to the release of refractoriness. Therefore, the mechanism by which α-ITC and β-ITC detect the auditory change in oddball sequences is similar to that of the N1 component. However, when deviant stimuli appear, the β-ITC responds earlier than N1. Haenschel et al. (2000) also found that the global field potential (GFP) in the β1 frequency band (12–20 Hz) increased earlier than the latency of MMN after the appearance of novel stimuli in hippocampal slices maintained *in vitro* and EEG monitoring in humans. Our results support the idea from the perspective of phase coherence that neural oscillations at β frequency are the earliest indicator of brain response to novel stimuli. In addition, when auditory stimuli contained midpoint frequency transition (DS3 and DS4), the N1-like latency of the second segment was significantly delayed, indicating that it was contaminated by the late ERP components generated by the first segment of the stimulus. However, the latency of β-ITC did not change significantly. Apparently, in detecting complex frequency changes within auditory objects, β-ITC may serve as a more stable and faster neural signal indicator compared to time-domain analysis results.

### 4.2. Characteristics of θ frequency in auditory change detection

In our study, the peak of θ-ITC in all deviants was significantly larger than that in the standard stimuli, demonstrating the auditory discriminative role of θ-ITC. Meanwhile, the larger time window of θ-ITC almost covers the latency of all components of the time-domain response, allowing it to integrate stimuli with different frequency modulation patterns into a single auditory representation and compare it with the existed memory traces (Naatanen and Winkler, 1999; Naatanen et al., 2007). The response characteristics of θ-ITC to auditory changes are similar to those of genuine MMN in time-domain analysis, which only appears when the memory trace formed by the standard stimulus is updated (Näätänen et al., 2011). The peak amplitude of the θ-ITC of DS3 was significantly higher than that of DS1, indicating that the standard frequency of the first part (DS3-1) effectively consolidated the memory trace, forming a distinct contrast with the subsequent deviant frequency (DS3-2). From another perspective, when the deviant stimulus differs from the standard stimulus in multiple feature dimensions simultaneously, the MMN amplitude induced by the deviant stimulus is approximately equal to the sum of the MMN induced by deviations in each dimension (Paavilainen et al., 2001; Wolff and Schroger, 2001). Therefore, in DS3, DS4, and DS5, in which multiple feature dimensions simultaneously violate the memory trace formed by the standard stimulus, the significantly increased phase-locking value at θ frequency may also result from the superposition of change-related responses. Thus, there is no significant difference in the peak of θ-ITC between DS3 and DS4, which have the same sum of deviant features compared to the Standard.

Previous research has confirmed that neural oscillation at the θ frequency is related to memory encoding and short-term memory maintenance (Klimesch et al., 2008). When incoming stimuli need to be compared with the previous one, significant phase resetting occurs at the θ frequency (Givens, 1996; Rizzuto et al., 2003). This is consistent with the memory trace hypothesis of MMN, which posits that incoming stimuli are compared with the memory trace formed by previous standard stimuli; when they do not match, the memory trace is refreshed and the MMN is generated (Näätänen et al., 2011; Fishman, 2014). According to our results, θ-ITC may be the neural basis for this comparison and updating process (Klimesch et al., 2006).

### 4.3. Comparison of α, β, and θ phase coherence

Even though ITC at β, α, and θ frequencies can discriminate deviant stimuli in oddball sequences, their underlying mechanisms might differ. Previous discussions on phase coherence and auditory change detection have mainly focused on the θ frequency. In these studies, the difference between deviant and standard stimuli involved either simple stimulus duration (Fuentemilla et al., 2008; Hsiao et al., 2009) or sound frequency differences (Ko et al., 2012; Choi et al., 2013), without considering internal frequency changes. Consequently, the phase-locking responses at different frequencies exhibited similar single peaks. Furthermore, lower oscillation rates in the θ band make it easier to achieve higher ITC. Therefore, when the ITC of the standard stimulus is subtracted from that of the deviant, the difference in θ frequency with a high phase coherence value is particularly significant. In contrast, changes in α and β bands, which have relatively higher frequencies but lower phase coherence values are easily overlooked. Our findings indicate that phase coherence at the α and β frequencies which have smaller response time windows can accurately differentiate frequency differences between deviant and standard stimuli. The θ band, on the other hand, can integrate the frequency and time feature of stimuli at a higher level and compare them with the memory traces formed by standard stimuli. In complex auditory environments, the ITC changes in multiple frequency bands work together to form a pre-attentive response to auditory changes. Correspondingly, in the time-domain analysis, the change-related response obtained by subtracting standard stimuli from deviant stimuli is the linear sum of the N1 wave differences and the genuine MMN (May and Tiitinen, 2010).

### 4.4. Characteristics of ITC induced by pure-tone and frequency-modulated stimuli

Additionally, we found that for DS 2 and DS 5-2 with complex time-frequency structures, ITC peak latencies at the β, α, and θ frequencies were longer compared to pure sinusoidal tone. According to the topographical distribution of frequency-specific neurons in the auditory cortex (Tiitinen et al., 1993; Schonwiesner and Zatorre, 2009; Saenz and Langers, 2014), processing sounds involving multiple frequencies requires the sequential activation of spatially widespread neurons, potentially necessitating more time for interregional communication and achieving higher phase coherence levels. The θ band can integrate multi-frequency features within a longer response time window, treating both DS-2 and DS5-2 as a single acoustic object. In real acoustic scenes, the slower modulation rate of the θ frequency enables syllable-scale temporal integration of speech information (Poeppel et al., 2008), whereas β and α can extract information within smaller time windows, sampling speech information with smaller linguistic grain sizes, such as phonemes (Giraud and Poeppel, 2012; Hamalainen et al., 2012). Consequently, although the detection of auditory changes may involve neural oscillatory activity across multiple frequencies, the observed neural oscillations may differ depending on the nature of the experimental stimuli.

There are some limitations to this study. First, although we have drawn comparisons between the response characteristics of the ITC at different frequencies to auditory change and the AERP components in the Oddball paradigm, we did not prove the causative link between them. Secondly, we didn’t investigate the spatial distribution of ITC at different frequencies and compare it with ERSP and time domain results. This aspect of the study could potentially provide valuable insights into the spatial patterns of neural responses to auditory stimuli, shedding light on how different frequency components are processed in the brain and contributing to a more comprehensive understanding of the auditory change detection process. Thirdly, the boundaries of each frequency band are inherently fuzzy. We did not conduct a detailed analysis based on individual peak frequencies and the differences in response to different stimuli. This nuanced examination would be crucial for a more refined understanding of how specific frequency components contribute to the auditory change detection process. In addition, despite the limitations of our study, such as participant age and hearing characteristics, our findings can be used to explore the impairment of auditory cognitive functions caused by aging and various neurological disorders. In fact, some researchers have recently focused on the changes of neural oscillatory synchrony in Parkinson’s disease (PD) and Alzheimer’s disease (AD) (Gallego-Jutgla et al., 2012; Karekal et al., 2022).

## 5. Conclusion

Our study identified the distinct roles of the ITC and ERSP across different frequencies in the auditory change detection process and revealed the unique characteristics of phase coherence in response to acoustic stimuli with different frequency change features. The findings not only deepen our understanding of the process of auditory change detection but also provide new insights and neural markers for the study of some neurological diseases.

## Data availability statement

The original contributions presented in this study are included in the article/supplementary material, further inquiries can be directed to the corresponding author.

## Ethics statement

The studies involving humans were approved by the Ethics Committee of Peking University First Hospital. The studies were conducted in accordance with the local legislation and institutional requirements. The participants provided their written informed consent to participate in this study.

## Author contributions

YL participated in the design of the study and revised the manuscript. CX collected the experimental data, performed the data analysis, and drafted the manuscript. JL designed the study and performed the data analysis, and RY and WS participated in the data collection. All authors have contributed to the manuscript and approved the submitted version.
